# Severe Infections in HIV-Exposed Uninfected Infants Born in a European Country

**DOI:** 10.1371/journal.pone.0135375

**Published:** 2015-08-18

**Authors:** Catherine Adler, Edwige Haelterman, Patricia Barlow, Arnaud Marchant, Jack Levy, Tessa Goetghebuer

**Affiliations:** 1 Pediatric Department, St Pierre University Hospital, Brussels, Belgium; 2 Clinical Investigation Unit, ImmuneHealth, Gosselies, Belgium; 3 Obstetrical Department, St Pierre University Hospital, Brussels, Belgium; 4 Institute for Medical Immunology, Université Libre de Bruxelles (ULB), Gosselies, Belgium; University of Cape Town, SOUTH AFRICA

## Abstract

**Background:**

Several studies indicate that HIV-exposed uninfected (HEU) children have a high infectious morbidity. We previously reported an increased incidence of group B streptococcus (GBS) infections in HEU infants born in Belgium.

**Methods:**

This study was undertaken to evaluate the incidence and risk factors of all cause severe infections in HEU infants born in Belgium between 1985 and 2006, including the pre-antiretroviral (ARV) prophylaxis era (1985 to 1994). The medical charts of 537 HEU infants followed in a single center were reviewed.

**Results:**

The incidence rate of severe infections during the first year of life was 16.8/100 HEU infant-years. The rates of invasive *S*. *pneumoniae* (0.62/100 infant-years) and GBS infections (1.05/100 infant-years) were, respectively, 4 and 13-fold higher in HEU infants than in the general infant population. Preterm birth was a risk factor for severe infections in the neonatal period (aOR = 21.34, 95%CI:7.12–63.93) and post-neonatal period (aHR = 3.00, 95%CI:1.53–5.88). As compared to the pre-ARV prophylaxis era, infants born in the ARV prophylaxis era (i.e., after April 1994) had a greater risk of severe infections (aHR = 2.93; 95%CI:1.07–8.05). This risk excess was present in those who received ARV prophylaxis (aHR 2.01, 95%CI 0.72–5.65) and also in those born in the ARV prophylaxis era who did not benefit from ARV prophylaxis as a result of poor access to antenatal care or lack of compliance (aHR 3.06, 95%CI 0.88–10.66).

**Conclusions:**

In HEU infants born in an industrialized country, preterm birth and being born during the ARV prophylaxis era were risk factors of severe infections throughout the first year of life. These observations have important implications for the clinical management of HIV-infected mothers and their infants.

## Introduction

Since 1996, administration of combined antiretroviral (cARV) agents in HIV-infected pregnant women has markedly reduced the risk of mother to child transmission (MTCT) of the virus [1], leading to an increasing number of HIV-exposed uninfected infants (HEU). The potential adverse effects of maternal HIV disease and of antenatal ARV prophylaxis on the outcome of the pregnancy and on the health of the infants have always been a concern. An association between the use of cARV during pregnancy and an increased risk of preterm delivery has been described in some studies [2]. The rate of congenital malformations was similar or slightly higher in HEU children compared to the general population but no association could be demonstrated with ARV exposure [2, 3]. Hematological toxicity has consistently been reported in HEU infants: cytopenia involving several lineages and persisting for several years was associated with the type and the duration of nucleoside reverse transcriptase inhibitors (NRTI) exposure [4–6], but no association with clinical complications has so far been reported.

Several publications from developing countries reported a high infectious morbidity and mortality risk in HEU infants compared to infants born to HIV-uninfected mothers [7–16]. In countries where the prevalence of HIV is high and access to prophylaxis of MTCT not generalized, the infectious mortality risk was correlated with advanced maternal HIV disease [8, 10, 12, 15]. Several studies have described an increased risk of hospitalizations, severe infections or infections caused by uncommon pathogens in HEU children [7, 10, 11, 13, 14, 17]. A large prospective study from Latin America reported a high incidence of low respiratory tract infection (LRTI) in HEU infants younger than 6 months [15]. We recently reported a significantly increased incidence of late onset invasive group B streptococcus (GBS) infection in HEU infants as compared to the general population of HIV-unexposed infants born in Belgium [18]. These observations led us to retrospectively examine the incidence and the risk factors of all severe infections occurring in 537 HEU children born to HIV-infected mothers between 1985 and 2006 and followed in our hospital. The inclusion of children born before the introduction of ARV prophylaxis allowed us to evaluate the occurrence of infection according to availability or exposure to ARV prophylaxis.

## Materials and Methods

### Setting of the study

This study was conducted at the Saint-Pierre hospital, a university-affiliated hospital with a large maternity department and a tertiary neonatal center, located in downtown Brussels, Belgium, and reference center for HIV-infected children and adults. Children born to HIV-infected mothers are followed at the pediatric outpatient clinic during the first 18 months of life in order to diagnose HIV transmission. A child is considered to be uninfected if RNA or DNA PCR is undetectable in at least 2 blood samples including one obtained after the age of 2 months or if a negative serology is documented. This study was approved by the hospital’s Ethics Committee.

### Measures to prevent MTCT of HIV

Preventive measures to reduce the risk of MTCT have been implemented over time as previously described [19]. All HIV-exposed children receive replacement feeding since 1985. ARV prophylaxis is recommended since 1994 (zidovudine (ZDV) monotherapy or ZDV and lamivudine (3TC) in mothers who delivered between April 1994 and 1997, and cARV afterwards) during at least the third trimester of pregnancy; intravenous (IV) ZDV is administered during delivery and orally to the newborn during the first 6 weeks of life since April 1994. After 1997, mothers already treated before delivery are continued on the same treatment throughout pregnancy [20]. Elective caesarean sections (CS) were recommended at 37 weeks of gestation, before onset of labor and rupture of membranes, between 1998 and 2006. As a significant number of infants born during the second time interval did not benefit from antenatal ARV prophylaxis, we defined a new variable combining period of birth and exposure to ARV during pregnancy. Interventions to prevent neonatal GBS invasive infections are implemented in the obstetric and neonatal departments according to international guidelines [21] adapted to local practice by the Belgian Superior Health Council [22] and were provided to all women.

### Data collection and clinical definitions

Maternal data included age, ethnicity, date of diagnosis of HIV infection, antenatal ARV prophylaxis, mode of delivery, plasma HIV viral load (VL) and CD4+ cell count at delivery (± 1 month). Plasma VL was measured with the Amplicor HIV-1 Monitor test (Roche, Switzerland) with a detection threshold of 400 copies/ml since 01/1996 and of 50 copies/ml since 2000. Newborn data included gestational age (GA), birth weight, and gender. The medical charts of all HEU infants were reviewed in order to identify hospitalizations for severe infections. Although these medical charts were not standardized, data were extracted in a standardized way from computerized hospitalizations’ reports for the purpose of the study.

During the neonatal period (before 29 days), as antibiotics are often started empirically, only episodes of microbiologically documented infections or those with laboratory markers of inflammation (C-reactive protein (CRP) > 20 mg/L) and/or clinical signs warranting IV antibiotic treatment for more than 5 days were recorded as severe infections. In the post-neonatal period (29–364 days of life), infections were defined as severe if IV antibiotic treatment was empirically initiated on admission because of suspected severe bacterial infection. For all hospitalizations for infection date of admission and discharge, final diagnosis, results of CRP, microbiological documentation and treatment were collected.

### Statistical analyses

The frequency of severe infection was computed according to baseline characteristics. As birth interval and exposure to ARV prophylaxis were strongly correlated they were combined in the analysis of baseline risk factors. Chi-square test and Fisher’s exact test were used to compare proportions, and Student’s t-test and Mann-Whitney U test to compare quantitative variables. Univariate and multivariate logistic regression analyses were performed to estimate crude and adjusted odds ratios (OR) for the associations between risk factors and the frequency of neonatal infections; ORs were adjusted for CD4 and preterm birth (n = 366). Cox proportional hazards models was used to estimate crude and adjusted hazard ratios (HR) for the associations between risk factors and the first occurrence of severe post-neonatal infection as well as with the occurrence of infection during the whole first year of life (repeated events were not taken into account). HRs were adjusted for maternal age, gender, and preterm birth (n = 446). Cases with missing data for any of the variables included in the model were dropped from the model. The risk of severe infection was estimated by Kaplan-Meier survival analysis censored at 1 year and for losses of follow up (FU), with log-rank test for significance. Statistical significance was assigned by a two-sided alpha level of 0.05. All *p-values* are two-tailed. Statistical analysis was performed using IBM SPSS Statistics version 20.

## Results

Between 1985 and 2006, 607 children were born alive to HIV-infected women and followed up in our center. Among them, 32 children were HIV-infected for 38 children no medical files could be found (among whom 23 born between January 1985 and April 12^th^ 1994). The study population includes 537 HEU children. The Table 1 presents the characteristics of the study population and the comparison of the children born at the time no ARV prophylaxis was recommended (first time interval: 1985 to April 12^th^ 1994, n = 82) with those born since ARV prophylaxis was recommended for the prevention of MTCT (second time interval: April 13^th^ 1994 to 2006, n = 455). Twenty eight mothers had repeat pregnancies including 10 who had a pregnancy in the first and in the second interval. The majority (83%) of the mothers originated from Sub Saharan Africa. The mean maternal age at delivery was 30 years, significantly higher during the second time interval. None of the mothers received ARV prophylaxis during the first time interval, and 36/447 (8.1%) of the mothers delivering after April 12^th^ 1994 did not benefit from ARV prophylaxis because of late presentation or non adherence. Delivery occurred by CS in 52% of the mother with a significantly higher rate during the second interval, mostly elective CS as recommended after 1998. The majority of the mothers were diagnosed with HIV infection before the pregnancy, but the diagnosis was made significantly later in the first time interval than in the second. Around delivery the median CD4+ cell count was 431 cells/mm^3^ (range 0–2350). VL was measured in 299 women who delivered after 1996, of whom 78% were undetectable. Preterm birth (< 37 weeks of gestation) occurred in 12% of the pregnancies, and 15% of the newborns weighed less than 2500 grams. Eighty four percent of the children were followed up for at least 1 year.

**Table 1:** Mothers and Neonates Baseline Characteristics by Period of Birth.

	Total N = 537	Born 01/10/1985-12/04/1994 N = 82	Born 13/04/1994-31/12/2006 N = 455	p-value
**MATERNAL CHARACTERISTICS**				
**African origin**				
** Yes**	423 (82.5%)	56 (74.7%)	367 (83.8%)	
** No**	90 (17.5%)	19 (25.3%)	71 (16.2%)	0.06
** Missing value**	24	7	17	
**Maternal age at delivery, years** *	467; 29.2 (5.4)	62; 27.0 (4.6)	405; 29.5 (5.4)	<0.0001^$^
**Maternal age at delivery, years**				
** 14–19**	18 (3.9%)	4 (6.5%)	14 (3.5%)	
** 20–24**	76 (16.3%)	15 (24.2%)	61 (15.1%)	
** 25–29**	150 (32.1%)	23 (37.1%)	127 (31.4%)	
** 30–34**	143 (30.6%)	17 (27.4%)	126 (31.1%)	
** 35–42**	80 (17.1%)	3 (4.8%)	77 (19.0%)	0.025
** Missing value**	70	20	50	
**Maternal CD4 cell count, last value before delivery, cells per mm** ^**3**^ **	378; 431 (0–2350)	46; 450 (0–2350)	332; 430 (0–1520)	0.87^£^
**Maternal CD4 cell count, last value before delivery, cells per mm** ^**3**^				
** <200**	49 (13.0%)	11 (23.9%)	38 (11.4%)	
** 200 to <500**	183 (48.4%)	16 (34.8%)	167 (50.3%)	
** ≥500**	146 (38.6%)	19 (41.3%)	127 (38.3%)	0.031
** Missing value**	159	36	123	
**Timing of HIV diagnosis**				
** Before pregnancy**	293 (63.4%)	31 (55.4%)	262 (64.5%)	
** During first trimester of pregnancy**	104 (22.5%)	8 (14.3%)	96 (23.6%)	
** During second trimester of pregnancy**	19 (4.1%)	5 (8.9%)	14 (3.4%)	
** During third trimester of pregnancy**	35 (7.6%)	6 (10.7%)	29 (7.1%)	
** Per or post partum**	11 (2.4%)	6 (10.7%)	5 (1.2%)	<0.0001^μ^
** Missing value**	75	26	49	
**ARV prophylaxis during pregnancy**				
** Yes**	490 (93.2%)	0 (0%)	408 (91.9%)	
** No**	36 (6.8%)	82 (100%)	36 (8.1%)	<0.001
**NEONATE’S CHARACTERISTICS**				
**Gender**				
** Male**	270 (50.3%)	44 (53.7%)	226 (49.7%)	
** Female**	267 (49.7%)	38 (46.3%)	229 (50.3%)	0.51
**Mode of delivery**				
** Vaginal**	251 (48.1%)	68 (84.0%)	183 (41.5%)	
** Caesarean section, elective**	198 (37.9%)	6 (7.4%)	192 (43.59%)	
** Caesarean section, emergency**	73 (14.0%)	7 (8.6%)	66 (15.0%)	<0.0001
** Missing value**	15	1	14	
**Gestational age at birth**				
** <37 weeks**	60 (11.6%)	8 (10.3%)	52 (11.9%)	
** ≥37 weeks**	456 (88.4%)	70 (89.7%)	386 (88.1%)	0.68
** Missing value**	21	4	17	
**Birth weight (grams)** *	526; 3037 (620)	80; 3154 (558)	446; 3016 (629)	0.07^$^
**Birth weight (grams)**				
** <2500**	80 (15.2%)	11 (13.8%)	69 (15.5%)	
** ≥2500**	446 (84.8%)	69 (86.2%)	377 (84.5%)	0.69
** Missing value**	11	2	9	
**Follow up time < one year**				
** Yes**	85 (15.8%)	13 (15.9%)	72 (15.8%)	
** No**	452 (84.2%)	69 (84.1%)	383 (84.2%)	1.00

* Number; mean (standard deviation)

** Number; median (min-max)

$ Student T test

£ Mann-Whitney test

μ Exact test (Fisher)

ARV = antiretroviral

### Incidence and risk factors for severe infections

Sixty-seven infants presented 81 episodes of severe infections during the first year of life (incidence rate: 16.8/100 infant-years). The details of these infections are presented in the supplementary table (S1 Table). Nine episodes of microbiologically documented invasive infections caused by encapsulated bacteria were observed in 8 infants (1.5%). GBS was isolated from the blood in 5 infants, 2 of them had early onset and 3 had late onset disease among whom one with a recurrent infection 38 days after a successful treatment, and one who died (S1 Table). The incidence rate of GBS infections was 1.05/100 infant-years. *S*. *pneumoniae* was isolated in the blood or CSF in 3 patients presenting with pneumonia, ethmoiditis and meningitis, resulting in an incidence rate of 0.62/100 infant-years of invasive *S*. *pneumoniae* infections.

Overall, 21 infants (4.0%) presented 22 episodes of severe infection during the neonatal period (first 28 days of life), and 52 infants presented 59 episodes of severe infections during the post-neonatal period (between 29 and 364 days of life). Among the neonatal infections, 4 were bacteremic infections caused by GBS, 3 by Staphylococcus *epidermidis*, 1 by Enterococcus *faecalis*, 1 by Candida *albicans*. For 13 episodes, no pathogen could be identified but laboratory markers of inflammation and/or clinical signs warranted antibiotic therapy for more than 5 days. The risk factors for severe neonatal infections are presented in Table 2. Preterm delivery was a strong independent risk factor. Maternal CD4+ cell count below 200/mm^3^ at delivery was associated with a two-fold increased frequency of severe neonatal infection but this association did not reach statistical significance. There was 1 severe neonatal infection during the first time interval (pre ARV prophylaxis era). Absence of antenatal ARV prophylaxis during the second interval conferred considerably higher risk of severe neonatal infection as compared with absence of antenatal ARV prophylaxis during the first interval (aOR = 11.03, 95%CI 1.15–105.57). Among the post-neonatal infections, almost half involved the respiratory system (46%), 15% the urinary tract, 14% the ENT system, 5% the neurological system, and less than 2% the digestive tract or the skin. Ten percent of the episodes requiring IV antibiotic on admission were fever without clinical focus in infants aged 29 days to 3 months and 7% occurred during the hospitalization in the neonatal intensive care unit after the 28^th^ day of life. The risk factors for severe post-neonatal infections are presented in Table 3. Preterm birth was significantly associated with post-neonatal infections. Maternal age above 35 years and male gender were other risk factors for post-neonatal infection.

**Table 2:** Frequency and Odds Ratios of Neonatal Severe Infection According to Baseline Characteristics (N = 519*, 21 Infants with Severe Neonatal Infection).

	n (%)	Crude odds ratio (confidence limits)	p-value^£^	Adjusted odds ratio** (confidence limits)	p-value^$^
**Maternal age at birth, years**					
** 14–34**	15 (4.0%)	1.00	-	1.00	-
** 35–42**	5 (6.2%)	1.60 (0.56–4.54)	0.38	1.64 (0.48–5.62)	0.43
**missing**	1				
**African origin**					
** No**	4 (4.7%)	1.00	-	1.00	-
** Yes**	16 (3.9%)	0.83 (0.27–2.55)	0.76	1.08 (0.25–4.61)	0.92
**missing**	1				
**Maternal CD4 cell count around birth, cells per mm** ^**3**^					
** ≥200**	12 (3.7%)	1.00	-	1.00	-
** <200**	4 (8.5%)	2.41 (0.74–7.81)	0.13	2.21 (0.59–8.28)	0.24
**missing**	5				
**Maternal plasma HIV-1 RNA around birth, copies/ml§**					
** <400**	6 (2.6%)	1.00	-	1.00	-
** ≥400**	7 (10.8%)	4.47 (1.45–13.80)	0.011	1.89 (0.47–7.55)	0.37
**missing**	8				
**Timing of HIV diagnosis**					
** Before or during first trimester of pregnancy**	15 (3.9%)	1.00	-	1.00	-
** Second or third trimester, per or post partum**	3 (4.7%)	1.22 (0.34–4.34)	0.73	0.84 (0.16–4.44)	0.84
**missing**	3				
**ARV during pregnancy**					
** No**	7 (6.4%)	1.00	-	1.00	-
** Yes**	14 (3.5%)	0.53 (0.21–1.34)	0.18	0.72 (0.22–2.42)	0.60
**Number of ARV during pregnancy**					
** None**	7 (6.4%)	1.00	-	1.00	-
** ZDV or ZDV/3TC**	2 (2.0%)	0.29 (0.06–1.45)	-	0.32 (0.03–3.13)	-
** cARV**	12 (4.0%)	0.61 (0.23–1.59)	0.29	0.83 (0.24–2.82)	0.62
**Duration of ARV during pregnancy**					
** Less than 3 months before delivery**	14 (5.4%)	1.00	-	1.00	-
** More than 3 months and less than 6 months**	2 (1.9%)	0.33 (0.08–1.50)	-	1.15 (0.21–6.36)	-
** More than 6 months**	4 (3.3%)	0.59 (0.19–1.84)	0.33	0.60 (0.15–2.49)	0.74
**missing**	1				
**Period of birth and ARV during pregnancy** #					
** Born 01 January 1985–12 April 1994**	1 (1.3%)	1.00		1.00	
** Born 13 April 1994–31 December 2006 & no ARV during pregnancy**	6 (18.2%)	16.67 (1.92–144.84)		11.03# (1.15–105.57)	
** Born 13 April 1994–31 December 2006 & ARV during pregnancy**	14 (3.5%)	2.71 (0.35–20.89)	0.001	3.12# (0.39–25.15)	0.045
**Gender**					
** Female**	12 (4.7%)	1.00		1.00	
** Male**	9 (3.4%)	0.73 (0.30–1.77)	0.49	0.83 (0.27–2.54)	0.74
**Mode of delivery**					
** Vaginal**	8 (3.3%)	1.00		1.00	
** Caesarean section, elective**	5 (2.6%)	0.76 (0.25–2.37)		0.73 (0.19–2.75)	
** Caesarean section, emergency**	8 (11.6%)	3.82 (1.38–10.59)	0.008	0.67 (0.16–2.86)	0.82
**Gestational age at birth**					
** ≥37 weeks**	8 (1.8%)	1.00	-	1.00	-
** ≤37 weeks**	13 (22.4%)	15.74 (6.20–40.01)	<0.0001	21.34 (7.12–63.93)	<0.0001

* Infants with a follow up <28 days were excluded.

** Adjusted for CD4 and preterm birth n = 366

£ Exact test (Fisher)

$ Wald test

§ Analysis restricted to period 1996–2006

# No adjustment for CD4 could be made (colinearity).

cARV = combined antiretroviral ZDV = zidovudine 3TC = lamivudine

**Table 3:** Hazard Ratios of Severe Infection Occuring Between 29 and 364 Days of Age According to Baseline Characteristics (N = 519*, 52 Infants with Severe Infections).

	n	Crude Hazard ratio (confidence limits)	p-value	Adjusted Hazard ratio** (confidence limits)	p-value
**Maternal age at birth, years**					
** 14–34**	30	1.00	-	1.00	-
** 35–42**	15	2.50 (1.35–4.65)	0.004	2.19 (1.16–4.13)	0.016
**missing**	6				
**African origin**					
** No**	9	1.00	-	1.00	
** Yes**	42	0.96 (0.47–1.97)	0.91	0.85 (0.40–1.80)	0.67
**missing**	1				
**Maternal CD4 cell count around birth, cells per mm** ^**3**^					
** ≥200**	33	1.00	-	1.00	
** <200**	6	1.28 (0.54–3.05)	0.58	1.46 (0.60–3.55)	0.40
**missing**	13				
**Maternal plasma HIV-1 RNA around delivery, copies/ml§**					
** <400**	25	1.00	-	1.00	-
** ≥400**	7	1.05 (0.45–2.42)	0.92	0.74 (0.31–1.80)	0.51
**missing**	20				
**Timing of HIV diagnosis**					
** Before or during first trimester of pregnancy**	41	1.00	-	1.00	-
** During second or third trimester, per or post partum**	5	0.71 (0.28–1.80)	0.48	0.63 (0.22–1.78)	0.38
**missing**	6				
**ARV during pregnancy**					
** No**	7	1.00	-	1.00	-
** Yes**	42	1.65 (0.74–3.68)	0.22	1.49 (0.62–3.57)	0.37
**missing**	3				
**Number of ARV during pregnancy**					
** None**	7	1.00	-	1.00	-
** ZDV or ZDV/3TC**	11	1.73 (0.67–4.46)	-	1.53 (0.53–4.43)	-
** cARV**	31	1.64 (0.72–3.73)	0.45	1.49 (0.61–3.64)	0.66
**missing**	3				
**Duration of ARV during pregnancy**					
** Less than 3 months before delivery**	21	1.00	-	1.00	-
** More than 3 months and less than 6 months**	12	1.43 (0.70–2.91)	-	1.52 (0.71–3.29)	-
** More than 6 months**	13	1.30 (0.65–2.60)	0.56	1.04 (0.50–2.16)	0.54
**missing**	6				
**Period of birth and ARV during pregnancy**					
** Born 01 January 1985–12 April 1994**	3	1.00	-	1.00	-
** Born 13 April 1994–31 December 2006 & no ARV during pregnancy**	4	3.52 (0.79–15.74)	-	1.66 (0.33–8.37)	-
** Born 13 April 1994–31 December 2006 & ARV during pregnancy**	42	2.80 (0.87–9.03)	0.19	1.86 (0.57–6.11)	0.59
**missing**	3				
**Gender**					
** Female**	17	1.00	-	1.00	-
** Male**	35	2.06 (1.16–3.69)	0.014	1.71 (0.92–3.19)	0.09
**Mode of delivery**					
** Vaginal**	20	1.00	-	1.00	-
** Caesarean section, elective**	19	1.17 (0.62–2.19)	-	0.90 (0.45–1.77)	-
** Caesarean section, emergency**	10	1.81 (0.85–3.86)	0.31	1.10 (0.46–2.64)	0.89
**Missing**	3				
**Gestational age at birth**					
** ≥37 weeks**	37	1.00	-	1.00	-
** <37 weeks**	13	3.00 (1.60–5.65)	0.001	3.00 (1.53–5.88)	0.001
**missing**	2				

* Infants with a follow up <28 days were excluded.

**Adjusted for maternal age, gender, and preterm birth n = 446

§ Analysis restricted to period 1996–2006

Data censored at 1 year of age. All p-values are from Wald tests.

cARV = combined antiretroviral ZDV = zidovudine 3TC = lamivudine

**Fig 1:**
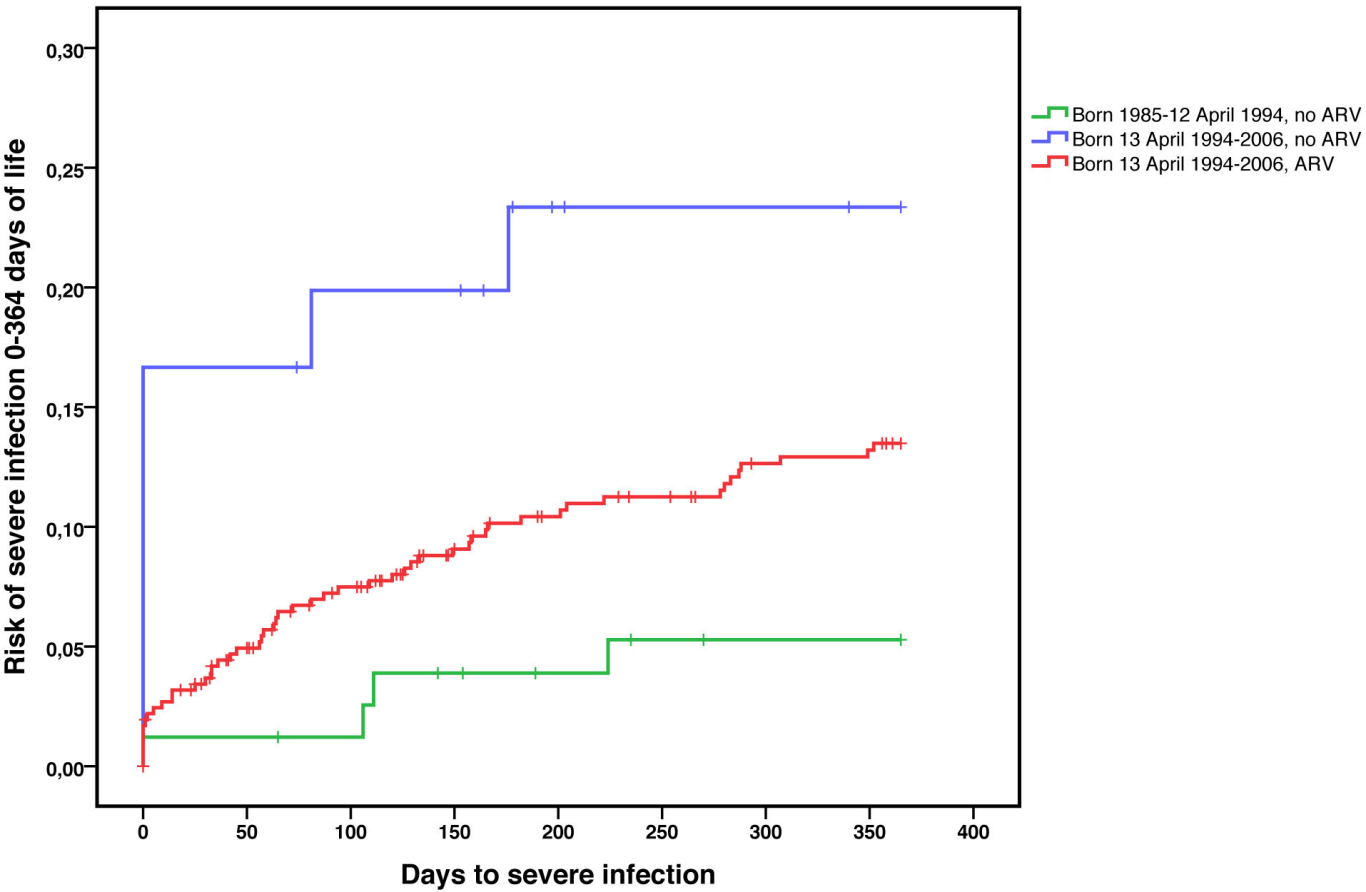
Occurrence of severe infections during the first year of life according to period of birth & exposure to ARV during pregnancy, Kaplan-Meier survival analysis (N = 537 infants).

When severe infections occurring over the whole first year of life were considered, the risk was significantly higher during the second time interval as compared to the first (HR = 2.93; 95%CI 1.07–8.05). The risk of severe infections was the highest in those not exposed to ARV prophylaxis and born in the second interval (HR = 5.63; 95%CI 1.69–18.70, aHR = 3.06; 95%CI 0.88–10.66), but it was also higher in infants born in the second interval and exposed to ARV (HR = 2.64; 95%CI 0.95–7.29, aHR = 2.01, 95%CI 0.72–5.65) as compared to infants born during the first study interval, all of them unexposed to ARV (Fig 1).

## Discussion

This large retrospective review of the medical history of HEU infants born between 1985 and 2006 and followed up in a single Belgian hospital identified an incidence rate of severe infections during infancy of 16.8/100 infant-years. The design of the study, retrospective and not controlled, did not allow to compare this rate to that of the general population, except for the rate of invasive infections caused by encapsulated bacteria. Invasive pneumococcal disease (IPD) over the first year of life occurred at a rate of 0.62/100 infant-years in this cohort, fourfold higher than the rate of 0.16/100 infants-years reported in the general population in Belgium before the introduction of the conjugated pneumococcal vaccine in 2005 (G. Hanquet, personal communication, [23]). A recent publication from South Africa reported an increased risk of IPD and mortality from IPD in HEU compared with HIV unexposed children, especially in young infants [24]. As previously described, the incidence rate of GBS infection was 13-fold higher in HEU infants from this cohort than in the general population (1.05 versus 0.08 per 100 infant-years) [18]. Recently a hospital-based surveillance study of invasive neonatal infections in South Africa has reported a 2.25-fold increased risk of neonatal invasive GBS infection in HEU compared to unexposed infants, particularly for late onset disease [16]. High infectious morbidity and mortality affecting HEU children have been reported repeatedly from developing countries [8, 12, 15]. In these studies the majority of infections involved the respiratory tract, as we observed in our cohort [12, 15]. Although the burden of infectious diseases affecting HEU children during the neonatal period has not yet been extensively studied, a high incidence of neonatal infections was observed in the prospective study of HEU infants born in Latin America but not in a study conducted in South Africa [25, 26].

In this study, preterm birth was significantly associated with infections occurring during the first year of life although the association was stronger for the neonatal period. Preterm birth is a known risk factor for neonatal infection and for hospitalization for infectious diseases until the age of 10 years [27, 28]. However the incidence of preterm birth in the study population (11.6%) was in the range reported in hospitals including a tertiary neonatal center in Belgium (11.8%) [29]. Independently from preterm birth, low maternal CD4+ cell count at delivery was associated, although not significantly, with an increased risk of infection particularly for the neonatal period. Several studies of HEU children born in developing countries indicated a correlation between the infectious mortality and morbidity and advanced maternal disease [10, 15, 25, 30]. This association was further supported by a recent multicentre retrospective study conducted in France and showing a significant association between low maternal CD4+ cell count and risk of bacterial infections in infancy [31]. Supporting the notion that maternal HIV disease interferes with infant’s immunity to infectious pathogens [32–36], several studies demonstrated a reduced transplacental transfer of antibodies from HIV-infected mothers as well as a reduced thymopoiesis and functional defects of antigen-presenting cells in HEU infants [35]. Formula feeding may also contribute to a reduction of infant’s immune defenses as absence of breastfeeding is associated with higher susceptibility to infections in early life [37]. Moreover maternal immunodeficiency may increase bacterial carriage.

Risk factors for post-neonatal infections include maternal age at delivery and male gender. Higher maternal age could be a marker of higher parity. As shown in other settings, a higher number of siblings may increase the risk of infection in HEU infants [25, 31], but unfortunately parity was not recorded in our study. Older maternal age may also be associated with a more advanced HIV disease. Male gender was a risk factor for post-neonatal infection. Male gender was associated with bacterial infection in HEU infants in the French study [31] and with susceptibility to infectious pathogens in other settings [38].

The results of study ACTG 076 demonstrating the benefit of ZDV to prevent MTCT were released in the first months of 1994. As soon as these results became available, the use of ZDV prophylaxis during pregnancy, at delivery and in the neonatal period became standard of care in our center. We show in this study that the risk of severe infection was higher in infants born after ARV prophylaxis recommendations had been issued. As some of the mothers of this group did not benefit from antenatal prophylaxis for various reasons including absence of follow up of the pregnancy, absence of diagnosis of maternal HIV infection, poor use of health care or poor adherence to medications, we sought to determine if the observed increased risk of infections was attributable to these infants. We therefore stratified the group of infants born after April 1994 for exposure to antenatal ARV prophylaxis and compared the 2 subgroups to the infants born before April 1994, all of them unexposed to ARV. The risk of severe infections was the highest in infants born after April 1994 and not exposed to ARV prophylaxis, particularly in the neonatal period, suggesting that this group is the most vulnerable. However we also found that infants born after April 1994 and who were exposed to ARV prophylaxis had a higher risk of severe infection than infants born earlier. Possible explanations for this association include exposure to ARV prophylaxis itself. Indeed, depressed hematopoiesis associated with exposure to NRTI in HEU infants may play a role, but this hypothesis could not be tested as differential leukocyte count was not analyzed in the present study. The association could also possibly be linked to differences in maternal characteristics between the two cohorts. Mothers in the earlier cohort were younger and, based on the history of the HIV epidemic, probably suffering from HIV infection for a shorter duration than mothers from the later cohort. However this hypothesis could not be tested since time of maternal infection and nadir CD4+ cell count were not available. Differences in maternal socioeconomic standing between the two time periods cannot be ruled out. The study population includes recent immigrants and families living often in poor socio-economic conditions. In Belgium, as in most part of the world, childhood mortality is associated with family income [39]. The role of these factors in the susceptibility of HEU infants to infections could not be evaluated in our study because socio-economic indicators were not available retrospectively.

The strengths of this study are its monocentric design, the analysis of data collected over a period of 20 years when major changes in the MTCT of HIV occurred, and the good follow up rate over the first year of life. However, the limitations of this study relate to the retrospective collection of data that were not recorded in a standardized way, resulting in a proportion of missing data and a number of potential confounders, including the socio-economic conditions, maternal diseases history and the number of siblings could not be analyzed. Moreover, some medical files could not be retrieved. However the follow up rate was similar between the two cohorts and an observational bias is unlikely as medical care was provided to HEU children by a pool of pediatricians unaware of a possible increased susceptibility to infectious diseases. Although the global risk excess of severe infections could not be quantified because of the absence of a control group, we reported an increased incidence rate of pneumococcal and GBS sepsis in HEU infants.

In conclusion, this retrospective study suggests that the increased susceptibility of HEU children to infectious diseases is not restricted to children born in developing countries. Preterm birth, maternal age and male gender were associated with incidence of severe infections during the neonatal and/or the post-neonatal periods. Moreover infants born in the ARV prophylaxis era had a greater risk of severe infections whether or not they were exposed to ARV prophylaxis. This increased incidence may be related to the characteristics of the mothers or involve the exposure to ARV agents. Prospective studies are urgently required worldwide to quantify the infectious risk in HEU infants and to determine the best clinical care and prevention that can be provided to this vulnerable population.

## Supporting Information

S1 Table Characteristics of the 67 children who presented a severe infection during the first year of life. (DOC)
