# Supplementary material for: Severe Infections in HIV-Exposed Uninfected Infants Born in a European Country
Source: PLoS One. 2015 Aug 18;10(8):e0135375. doi: 10.1371/journal.pone.0135375 (PMC4540431; doi:10.1371/journal.pone.0135375)
Supplement: S1 Table — (DOC) [file pone.0135375.s001.doc]

SUPPLEMENTARY TABLE 1: CHARACTERISTICS OF THE 67 CHILDREN WHO PRESENTED A SEVERE INFECTION DURING THE FIRST YEAR OF LIFE

| ID | Birth year | sex | GA | BW | Mat CD4 (cell /mm³) | ARV exposure | Age at infection (days) | Type of infection | isolate | | CRP (mg/L) | antibiotic duration (days) |
| --- | --- | --- | --- | --- | --- | --- | --- | --- | --- | --- | --- | --- |
| 1 | 1990 | F | 37 | 2260 | 70 | 0 | 106 | LRTI | *RSV* | missing | | missing |
|  |  |  |  |  |  |  | 172 | LRTI | *-* | missing | | missing |
| 2 | 1992 | F | 38 | 3070 |  | 0 | 111 | UTI | *E.coli* | missing | | 10 |
|  |  |  |  |  |  |  | 336 | LRTI | *-* | missing | | missing |
| 3 | 1994 | M | 38 | 2890 | - | 0 | 0 | MF | *-* | missing | | 7 |
| 4 | 1994 | M | 35 | 3200 | - | 0 | 224 | LRTI | *S.pneumoniae* | missing | | missing |
| 5 | 1995 | M | 40 | 3590 | 240 | ZDV ± 3TC | 157 | LRTI | *-* | missing | | missing |
| 6 | 1995 | M | 38 | 3300 | 890 | 0 | 77 | LRTI | *-* | missing | | missing |
| 7 | 1996 | M | 41 | 4500 | 440 | ZDV ± 3TC | 30 | UTI | *E.coli* | | 38 | 7 |
| 8 | 1996 | M | 41 | 2830 | 840 | ZDV ± 3TC | 0 | MF | *-* | | 38 | 10 |
| 9 | 1996 | F | 33 | 1440 | 840 | ZDV ± 3TC | 65 | FWF | *Rhinovirus* | | 38 | 2 |
| 10 | 1996 | M | 40 | 3230 | 430 | 0 | 176 | LRTI | *-* | missing | | missing |
| 11 | 1996 | F | 37 | 3915 | 880 | 0 | 0 | MF | *-* | | 17 | 7 |
| 12 | 1997 | M | 37 | 3205 | 170 | ZDV ± 3TC | 352 | LRTI | *-* | missing | | missing |
| 13 | 1998 | M | 39 | 3870 | - | ZDV ± 3TC | 287 | LRTI | *-* | missing | | 4 |
|  |  |  |  |  |  |  | 322 | LRTI | *-* | | 162 | 7 |
| 14 | 1998 | M | 39 | 3570 | - | ZDV ± 3TC | 280 | ethmoiditis | *-* | missing | | missing |
| 15 | 1998 | M | 37 | 1900 | 940 | ZDV ± 3TC | 81 | LRTI | *-* | | 0 | 3 |
| 16 | 1998 | M | 35 | 2310 | - | cARV | 0 | MF | *-* | missing | | 7 |
| 17 | 1998 | M | 40 | 3050 | 290 | ZDV ± 3TC | 158 | LRTI | *-* | missing | | 4 |
| 18 | 1998 | M | 37 | 2560 | 710 | ZDV ± 3TC | 87 | Acute otitis | *S.pneumoniae* | | 154 | 5 |
| 19 | 1998 | M | 38 | 3300 | 558 | ZDV ± 3TC | 133 | AGE | *Salmonella* | missing | | missing |
| 20 | 1999 | F | 38 | 2900 | 273 | cARV | 36 | otitis | *-* | | 47 | 7 |
| 21 | 1999 | M | - | - | - | - | 164 | UTI | *-* | missing | | 5 |
| 22 | 1999 | M | 33 | 1700 | 217 | cARV | 0 | MF | *-* | | <1 | 7 |
| 23 | 1999 | M | 38 | 2900 | - | ZDV ± 3TC | 0 | MF | *-* | missing | | 7 |
|  |  |  |  |  |  |  | 34 | meningitis | *-* | | 82 | 6 |
| 24 | 1999 | M | 38 | 3390 | 132 | cARV | 307 | UTI | *K. pneumoniae* | | 63 | 7 |
|  |  |  |  |  |  |  | 328 | UTI | *P. aeruginosa* | | 105 | 7 |
| 25 | 2000 | M | 38 | 3510 | 1100 | cARV | 14 | UTI | *S. epidermidis* | missing | | 7 |
| 26 | 2000 | M | 33 | 2900 | - | cARV | 0 | LRTI | *-* | missing | | 12 |
| 27 | 2000 | M | 30 | 995 | 295 | cARV | 149 | UTI | *E.coli* | | 28 | 5 |
| 28 | 2000 | F | 34 | 2920 | 259 | cARV | 182 | LRTI | *-* | | 75 | 2 |
| 29 | 2001 | F | 33 | 2360 | 433 | 0 | 0 | sepsis | *GBS** | | 61 | 10 |
| 30 | 2001 | M | 24 | 630 | 744 | 0 | 0 | MF | *-* | | 23 | 7 |
| 31 | 2001 | M | 38 | 2900 | 1213 | cARV | 64 | meningitis | *Enterovirus* | | 9 | 3 |
| 32 | 2002 | M | 38 | 3000 | 440 | cARV | 94 | UTI | *-* | missing | | 10 |
| 33 | 2002 | F | 38 | 3300 | 274 | cARV | 45 | LRTI | *-* | missing | | missing |
| 34 | 2002 | M | 31 | 1350 | 104 | cARV | 72 | sepsis | *GBS** | missing | | 21 |
| 35 | 2002 | M | 37 | 2650 | 39 | cARV | 0 | LRTI |  | missing | | 14 |
| 36 | 2002 | M | 39 | 2900 | 412 | cARV | 349 | LRTI | *RSV* | | 10 | missing |
| 37 | 2002 | F | 37 | 2600 | 505 | cARV | 109 | LRTI | *RSV* | | 153 | 4 |
| 38 | 2003 | M | 33 | 1380 | 859 | cARV | 2 | LRTI | *-* | | 12 | 7 |
|  |  |  |  |  |  |  | 143 | mastoiditis | *-* | missing | | missing |
| 39 | 2003 | M | 38 | 2710 | 202 | cARV | 201 | ethmoiditis | *S.pneumoniae** | | 105 | 11 |
| 40 | 2003 | M | - | 3090 | - | cARV | 120 | LRTI | *-* | | - | missing |
| 41 | 2003 | M | 29 | 950 | 108 | 0 | 0 | MF | *-* | | 15 | 10 |
|  |  |  |  |  |  |  | 40 | NEC | *E. fecalis** | | 112 | 17 |
| 42 | 2003 | M | 40 | 3500 | - | - | 30 | LRTI | *-* | missing | | missing |
| 43 | 2003 | F | 33 | 1700 | 480 | cARV | 33 | meningitis | *GBS** | | 277 | 5 |
| 44 | 2003 | F | 38 | 2830 | 373 | cARV | 25 | sepsis | *GBS** | | 111 | 12 |
|  |  |  |  |  |  |  | 74 | sepsis | *GBS** | | 8 | 6 |
| 45 | 2003 | M | 38 | 3000 | 202 | cARV | 63 | sepsis | *S.pneumoniae** | | 208 | 7 |
| 46 | 2004 | M | 40 | 3380 | 346 | cARV | 278 | LRTI | *-* | missing | | missing |
| 47 | 2004 | M | 40 | 3310 | 503 | cARV | 166 | ethmoiditis | *S.pneumoniae* | | 18 | 7 |
| 48 | 2004 | F | 26 | 825 | 15 | cARV | 14 | MF | *-* | | 4 | 5 |
| 49 | 2004 | F | 26 | 805 | 605 | 0 | 0 | sepsis | *E. fecalis** | | <3 | 8 |
|  |  |  |  |  |  |  | 113 | FWF | *Parainfluenza* | | 1 | 3 |
| 50 | 2004 | F | 31 | 1170 | 206 | cARV | 42 | NEC | *-* | | <1 | 7 |
| 51 | 2004 | F | 32 | 1915 | 22 | cARV | 0 | MF | *-* | | 4 | 7 |
| 52 | 2004 | F | 36 | 2955 | - | 0 | 0 | sepsis | *GBS* | | 180 | 10 |
| 53 | 2004 | F | 39 | 2545 | 802 | cARV | 57 | otitis | *-* | | 10 | 10 |
|  |  |  |  |  |  |  | 129 | LRTI | *-* | | 28 | 2 |
| 54 | 2005 | M | 38 | 2600 | 543 | cARV | 9 | meningitis | *GBS** | | 204 | 16 |
| 55 | 2005 | F | 40 | 3820 | 418 | cARV | 165 | meningitis | *S.pneumoniae** | | missing | 11 |
| 56 | 2005 | M | 28 | 940 | 764 | cARV | 0 | MF | *-* | | 4 | 7 |
|  |  |  |  |  |  |  | 24 | NEC | *-* | | <1 | 7 |
|  |  |  |  |  |  |  | 39 | NEC | *-* | | <1 | 7 |
| 57 | 2005 | M | 40 | 3740 | 494 | cARV | 283 | LRTI | *-* | | 43 | 6 |
| 58 | 2005 | F | 37 | 3110 | - | missing | 35 | cellulitis | *-* | | 4 | 5 |
| 59 | 2005 | M | 33 | 1790 | 190 | cARV | 58 | FWF | *-* | | 28 | 3 |
| 60 | 2005 | F | 38 | 3110 | 300 | cARV | 204 | LRTI | *-* | | 131 | 3 |
| 61 | 2005 | M | 35 | 3880 | 275 | cARV | 5 | sepsis | *S. epidermidis** | | 68 | 7 |
| 62 | 2005 | M | 38 | 2930 | 355 | cARV | 56 | FWF | *-* | | 42 | 5 |
| 63 | 2006 | F | 39 | 3570 | - | cARV | 288 | UTI | *E.coli* | | 72 | 5 |
| 64 | 2006 | M | 37 | 2360 | - | cARV | 222 | LRTI | *Enterovirus* | | 59 | 2 |
| 65 | 2006 | M | 35 | 2460 | - | cARV | 129 | FWF | *-* | | 48 | 5 |
| 66 | 2006 | F | 38 | 3550 | 449 | cARV | 126 | UTI | *E.coli* | | 152 | 5 |
| 67 | 2007 | M | 38 | 3310 | - | cARV | 33 | FWF | *-* | | 7 | 3 |
|  |  |  |  |  |  |  | 348 | LRTI | *-* | | 140 | 3 |

*isolated in the blood and/or CSF

cARV= combined antiretroviral, ZDV= zidovudine, 3TC= lamivudine, NEC= necrotizing enterocolitis, LRTI= low respiratory tract infection, FWF= fever without focus, MF= maternofetal, UTI= urinary tract infection
